# Complete submergence triggers synergistic regulation of gibberellin-abscisic acid balance and pith cavity development to promote stem elongation in *Alternanthera philoxeroides*

**DOI:** 10.3389/fpls.2025.1694732

**Published:** 2025-11-17

**Authors:** Shufang Jing, Hongwei Liu, Yuezhen Li, Zhenxia Bai, Mingfu Yu, Junhe Liu, Dahong Li

**Affiliations:** School of Biological Science and Food Engineering, Huanghuai University, Zhumadian, China

**Keywords:** abscisic acid, *Alternanthera philoxeroides*, gibberellin, physiological response, pith cavity, stem elongation, submergence stress

## Abstract

Submergence, a major abiotic stress in hydrologically dynamic ecosystems, poses severe challenges to plant survival and growth. Existing studies have demonstrated that plants employ a suite of adaptive strategies to tolerate submergence. These divergent adaptive responses are endogenously regulated by phytohormones; yet, the underlying mechanisms that connect hormonal regulation, anatomical plasticity, and growth adaptation in the context of submergence remain insufficiently elucidated. *Alternanthera philoxeroides* (Mart.) Griseb. is widely distributed in disturbed, flood-prone habitats and exhibits exceptional adaptability to hydrological fluctuations, making it a suitable species for exploring submergence stress responses. This study investigated *A. philoxeroides’* responses to three hydrological conditions (non-submergence, partial submergence, complete submergence), focusing on stem growth and its anatomical and hormonal regulatory drivers. Results revealed an unexpected growth pattern: complete submergence induced significantly faster stem elongation than partial submergence, with this growth-promoting effect most pronounced in immature stems—particularly the basal parts of immature internodes. This elongation correlated positively with enlarged pith cavities and elevated gibberellin (GA_4_), while it was significantly negatively correlated with abscisic acid (ABA). GA_4_ content and pith cavity area were also highly positively correlated. These findings unravel a critical adaptation mechanism in *A. philoxeroides*: coordinated hormonal adjustments (GA_4_ up, ABA down, higher GA_4_/ABA) and morphological remodeling (pith cavity enlargement) that synergistically support enhanced growth under severe submergence. This work advances understanding of plant adaptive strategies under climate-driven hydrological stress, enriches insights into abiotic stress response mechanisms, and provides valuable references for wetland ecosystem conservation and the improvement of crop submergence tolerance.

## 1 Introduction

Abiotic stresses are major constraints on plant growth and ecosystem function, with hydrological fluctuations—especially submergence—emerging as a critical threat in wetland, riparian, and agricultural ecosystems globally (Fukao et al., 2019; Ganguli et al., 2020; Milly et al., 2002). As climate change intensifies the frequency and duration of flooding events, plants in these habitats face heightened pressure to adapt to prolonged or intermittent submergence (Bejarano et al., 2018; Bertola et al., 2020). For wetland-associated species, the ability to maintain growth and survival under submergence is not only vital for their own persistence but also for the stability of ecological processes like nutrient cycling and habitat provision (Lehner et al., 2011; León et al., 2021; Souza et al., 2021). Plant growth strategies in response to submergence are highly context-dependent, with distinct adaptive trajectories shaped by the degree of submergence—specifically partial submergence (where aerial tissues remain exposed) and complete submergence (where the entire plant is underwater) (Ashikari et al., 2025; Bailey-Serres and Voesenek, 2008). However, the mechanisms by which plants coordinate physiological and morphological adjustments to cope with submergence remain incompletely understood, particularly for species that thrive in hydrologically dynamic environments (Pan et al., 2023).

Plants have evolved diverse strategies to tolerate submergence, among which hormonal regulation and anatomical plasticity are two well-documented pillars (Ashikari et al., 2025; Rankenberg et al., 2021; Yeung et al., 2018). Hormones, such as gibberellins (GAs), play pivotal roles in mediating growth responses to submergence—for instance, driving stem elongation to reach the water surface and restore gas exchange (Ayano et al., 2014; Jin et al., 2017). However, GAs and abscisic acid (ABA) often act as antagonists to fine-tune growth under submergence (Wang and Komatsu, 2022). GAs promote stem elongation to escape submergence (e.g., by upregulating cell wall loosening genes), while ABA typically restrains excessive elongation and prioritizes stress tolerance (Jin et al., 2017). This dynamic GA-ABA balance is emerging as a critical determinant of how plants allocate energy between growth and stress resilience (Cox et al., 2004). Meanwhile, anatomical traits like aerenchyma (e.g., pith cavity, gas-filled structures in stems or roots) enhance hypoxia tolerance by facilitating the diffusion of oxygen and other gases between aerial and submerged tissues (Fan et al., 2015; Yang et al., 2019). While individual studies have explored GA-mediated growth or aerenchyma formation under submergence, the regulatory links between these two traits—i.e., how plant hormones and anatomical plasticity interact across varying degrees of submergence (partial *vs*. complete)—remain substantially underexplored (Daniel and Hartman, 2024). This knowledge gap is particularly critical given the context-dependent nature of plant submergence strategies: the hormone-anatomy interactions that facilitate escape under partial submergence may differ fundamentally from those supporting quiescence under complete submergence. Filling this gap via case studies of flood-adapted species is essential to unraveling the integrated adaptive networks that enable plants to cope with the full spectrum of hydrological stress, rather than isolated traits in single submergence scenarios (Ashikari et al., 2025).

*Alternanthera philoxeroides* (Mart.) Griseb. is a typical wetland species widely distributed in disturbed, flood-prone habitats, including freshwater marshes, riverbanks, and agricultural ditches (Huang et al., 2025; Tanveer et al., 2018). A hallmark of this species is its remarkable adaptability to hydrological variability—particularly submergence: it can thrive and persist under non-submerged conditions, partial submergence, and even complete submergence (Ayi et al., 2016; Fan et al., 2015).This high hydrological flexibility makes it a suitable species for investigating submergence adaptation mechanisms. Despite its ecological relevance, however, little is known about how *A. philoxeroides* coordinates hormonal dynamics and anatomical changes to support growth under submergence. Specifically, whether GAs are involved in regulating aerenchyma formation in its stems, and how this coordination affects growth performance under different submergence regimes, remains unaddressed.

To fill this critical gap in understanding how plant hormones and anatomical plasticity coordinate to mediate submergence adaptation across variable hydrological gradients, the present study exposed *A. philoxeroides* to three hydrological treatments: non-submergence, partial submergence, and complete submergence. We focused on stem growth as a key indicator of submergence adaptation—given that stem elongation constitutes a primary strategy for plants to escape submergence-induced hypoxia. Our specific objectives were: (1) to characterize the stem growth responses of *A. philoxeroides* to these distinct submergence conditions; (2) to quantify changes in stem anatomical traits (with a focus on pith cavity development) and GA_4_ levels (a bioactive gibberellin) under each treatment—plus abscisic acid (ABA, a stress-responsive hormone) as a complementary indicator for GA_4_’s regulatory role; (3) to explore correlations between GA_4_ content (with ABA content as a complementary reference), pith cavity development, and stem growth rate, thereby testing the hypothesis that GA_4_ and aerenchyma formation act synergistically to support submergence adaptation. This study, by unraveling hormonal-anatomical interplay in submerged *A. philoxeroides*, aims to advance understanding of flood adaptation—addressing the critical gap in their coordinated role across hydrological gradients. Findings will clarify the species’ ecological success, inform wetland species’ tolerance research, and support wetland management and crop (e.g., rice) flood tolerance improvement under climate change.

## 2 Materials and methods

### 2.1 Plant material and cultivation

*Alternanthera philoxeroides* (Mart.) Griseb., a perennial herbaceous species with strong adaptability and competitiveness, was used to investigate the effects of submergence on plant growth. Experimental materials were prepared following our previous study (Jing et al., 2022). Young stem cuttings were used for propagation to minimize maternal and environmental effects on growth. Cuttings were collected in May 2024 from a natural population along the Qinghe River bank, Zhumadian City, Henan Province (32°18’ N, 113°11’ E). ~20 cm-long cuttings were planted in pots (13 cm in depth and diameter) at the Experimental Station of Huanghuai University, Zhumadian (32°18’ N, 113°11’ E). Cultivated under uniform natural conditions in an open-air setting, plants were used for experiments ~1 month after cultivation when their stems reached 30 cm with 12 internodes.

### 2.2 Experimental design

Three experimental treatments were conducted under complete darkness to eliminate light interference, with 20 plants per group: non-submergence, partial submergence, and complete submergence. All groups were placed in a dark laboratory. For the non-submergence group, plants were watered routinely to maintain the soil at field capacity, with no submergence occurring during the treatment period. For the partial submergence group, plants were placed in water tanks (approximately 60 cm in height), where water levels were controlled to submerge the lower half of each plant’s stem—ensuring consistent submergence depth throughout the treatment period. For the complete submergence treatment, plants were placed in 1.2 m high water tanks, with water maintained to fully submerge the plants and maintain a 50 cm water layer above the top of each plant, ensuring no aerial parts were exposed.

To prevent light from interfering with plant growth, green light lamps were selected and used as auxiliary light sources throughout the entire treatment period—this choice was based on the fact that plants absorb less green light. These lamps facilitated measurement, observation, and recording when such operations were conducted. Since plants in the complete submergence group might produce gaseous substances (e.g., ethylene) during treatment, measurements were performed underwater to prevent plants from emerging. After measurement, plants were immediately re-submerged to the original depth to continue treatment.

Slow aeration was applied via air pumps at 8:00 AM and 6:00 PM daily to ensure sufficient and uniform O_2_ and CO_2_ supply in both partial and complete submergence treatments. Dissolved oxygen, temperature, and pH of water in different tanks were measured twice daily (8:00 AM and 8:00 PM) using a multi-parameter water quality analyzer (Hydrolab DS5, Hach, USA). No significant differences in these parameters were observed among treatments during the experiment (**Table 1**).

**Table 1:** Physicochemical properties of water in tanks during the experiment.

Treatments	Temperature (°C)	pH	Dissolved oxygen concentration (mg L^-1^)
Non-submergence	25.44 ± 0.01 a	–	–
Partial submergence	25.43 ± 0.01 a	7.02 ± 0.06 a	8.02 ± 0.07 a
Complete submergence	25.41 ± 0.01 a	7.04 ± 0.07 a	8.04 ± 0.06 a

The temperature, pH, and dissolved oxygen concentration of the water at different tanks were measured twice daily (8:00 AM and 8:00 PM) using a multi-parameter water quality analyzer (Hydrolab DS5, Hach, USA) during the experiments. Data are means ± SE, *n* = 48 (total measurements of treatments); “-” indicates no data available. Values with the same lowercase letter are not significantly different (temperature: Duncan’s test; pH/DO: independent t-test; *p* > 0.05).

### 2.3 Detection of plant growth parameters

Prior to submergence treatments, the first basal, median, and uppermost visible internodes of the main stem in *A. philoxeroides* were marked with red fine polyester thread following Jing et al. (2024a). This marking helped distinguish mature from immature internodes. Stem length and internode lengths at different positions were measured with a ruler. For each group, the stem length of 20 plants was measured every 2 days to monitor growth dynamics.

Based on preliminary experimental observations, the fastest-growing immature internode was selected and marked. This internode was then divided into three equal segments, which were further marked with thin threads to distinguish its basal, middle, and upper parts.

### 2.4 Detection of plant anatomical indices

For plants harvested at 0 days (i.e., before submergence initiation) and those with marked internodes post different submergence treatments (which were sampled at the end of submergence treatments), stem samples were collected from each marked segment, from which 1 cm-long sections were excised with a sterile blade to avoid internal structural damage. Transverse sections were then prepared from the base of the marked internodes, with three biological replicates per treatment group (Jing et al., 2024a). Images of these sections were captured using a stereomicroscope imaging system (SMZ25, Nikon, Japan) and saved in TIFF format. Stem wall thickness and pith cavity area were measured using Nikon NIS-Elements BR software (version 4.30).

### 2.5 Methods for hormone detection

Basal parts of mature and immature internodes were selected as experimental samples. These basal parts were transversely excised with a double-edged blade, quickly placed into labeled plastic cryogenic tubes, immediately flash-frozen in liquid nitrogen, and then stored at -80 °C until hormone assay. For hormone determination, basal parts of mature internodes from the 4 plants were pooled as one biological replicate; similarly, basal parts of immature internodes from the same 4 plants were pooled as one biological replicate. Each treatment included 3 biological replicates for hormone analysis. GA_4_ and ABA concentrations were measured by HPLC-MS/MS. The HPLC-MS/MS system was composed of a high-performance liquid chromatography (HPLC, Agilent Technologies 1200 series, USA) coupled to an AB Sciex API 6500 Qtrap mass spectrometer (Concord, ON, Canada). Analyst 1.6.3 software (Concord, ON, Canada) was used to control the HPLC-MS/MS system, while the Multiquant 3.2 software was employed for data processing; detailed procedures were performed as described in our previous study (Jing et al., 2024b; Pan et al., 2010).

### 2.6 Data analysis


Elongation of stem(cm)=Stem length after treatment−Stem length before treatment;

Elongation of mature stem(cm)=Mature stemlength after treatment−Mature stemlength before treatment;

Elongation of immature stem(cm)=Immature stemlength after treatment−Immature stemlength before treatment.

Hormone concentration in original samples was calculated from the detected concentration of the final extract (measured by the instrument) using the following formula (Pan et al., 2010):


$$\text{Original sample hormone concentration }(\text{ng }/\text{ g})=\frac{\text{Detected concentration}(\text{ng }/\text{ ml})\times \text{Dilution volume}(\text{ml})}{\text{Weighed mass}(\text{g})}$$

where dilution volume is the volume of solution for final dissolution and injection, and weighed mass is the mass of sample used for extraction. Detected concentration was automatically calculated by the instrument via substituting the target substance’s peak area into the standard curve equation.

Prior to statistical analysis, all data (including elongation of stem, mature/immature stem; number of newly formed internodes; elongation of basal/middle/upper parts of marked internodes; stem wall thickness; pith cavity area; and GA_4_ and ABA concentrations) were tested for normality(Shapiro-Wilk test) and variance homogeneity (Levene’s test). All hormone and growth trait data passed normality tests, W > 0.90, *p* > 0.05; Levene’s test confirmed homogeneous variance, F < 1.5, *p* > 0.05. One-way analysis of variance (one-way ANOVA) was used to compare these parameters across submergence treatments, followed by Duncan’s multiple range test for *post-hoc* comparisons of inter-treatment differences. Pearson correlation analysis was performed to examine correlations between immature stem elongation and the following variables: pith cavity area, stem wall thickness, GA_4_ concentration, ABA concentration, GA_4_/ABA, and elongation of the basal, middle, and upper parts of marked internodes. All analyses were carried out using SPSS Statistics 26.0 software (IBM Corp., Armonk, NY, USA). Graphs were generated using Origin 2021 software (OriginLab, Northampton, MA, USA), with statistical significance determined at *p* < 0.05.

## 3 Results

### 3.1 Stem growth

**Figure 1:**
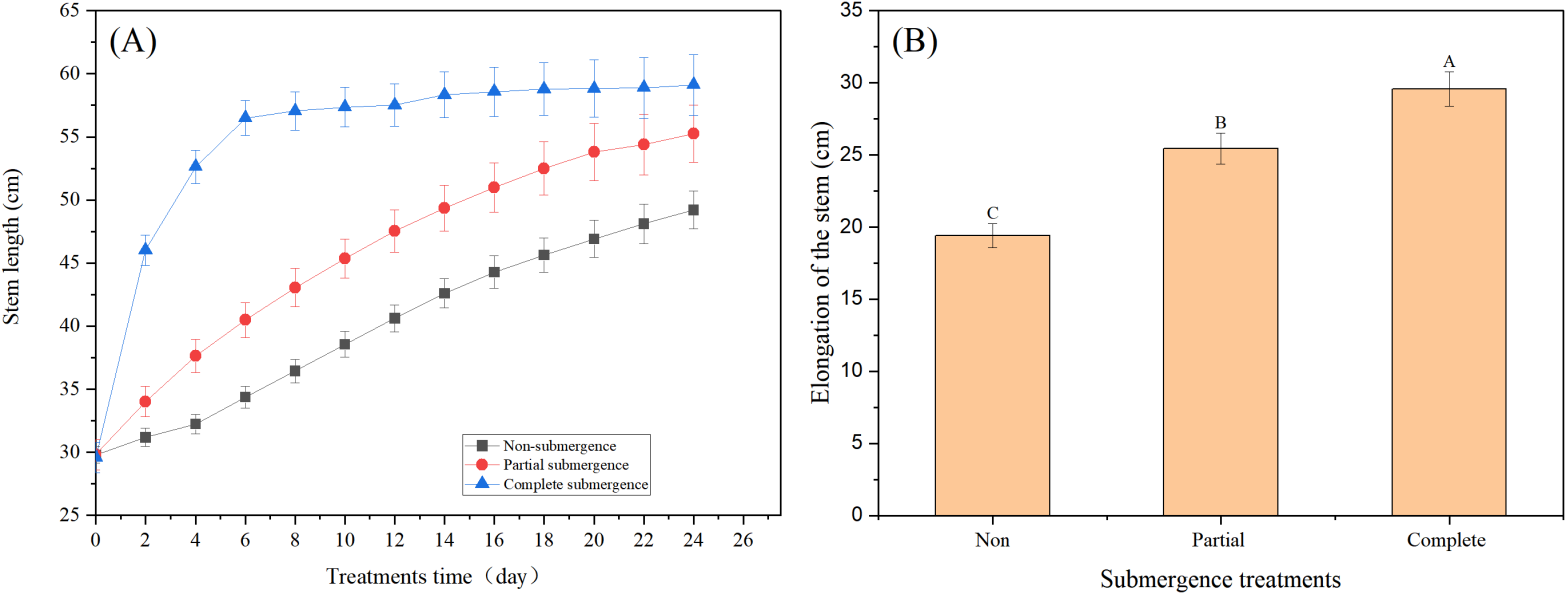
Stem length of *Alternanthera philoxeroides* under different treatment environments at different treatment times **(A)** and stem elongation at the end of treatment **(B)**. Data are means ± SE, *n* = 20 (biological replicates,each representing an independent plant). Different letters indicate significant difference between treatments (Duncan’s test, *p* < 0.05).

Before the experiment, no significant difference in stem length was observed among all treatment groups (*p* > 0.05, **Figure 1A**). After treatment initiation, the non-submergence and partial submergence groups exhibited slow growth, while the complete submergence group showed abrupt rapid growth, which shifted to steady growth after 6 days and persisted until the experiment termination (**Figure 1A**). Compared to the stem lengths before treatment, the mean total stem elongation was 19.40 cm in non-submergence group, 25.44 cm under partial submergence, and 29.56 cm under complete submergence (**Figure 1B**). Statistical analysis indicated that both submergence treatments significantly increased stem elongation compared to the non-submergence group (*p* < 0.05, **Figure 1B**); additionally, complete submergence resulted in significantly greater elongation than partial submergence (*p* < 0.05, **Figure 1B**). These results indicate that both partial and complete submergence promoted stem elongation in *A. philoxeroides*, with the promoting effect more pronounced under complete submergence.

### 3.2 Internode growth

**Figure 2:**
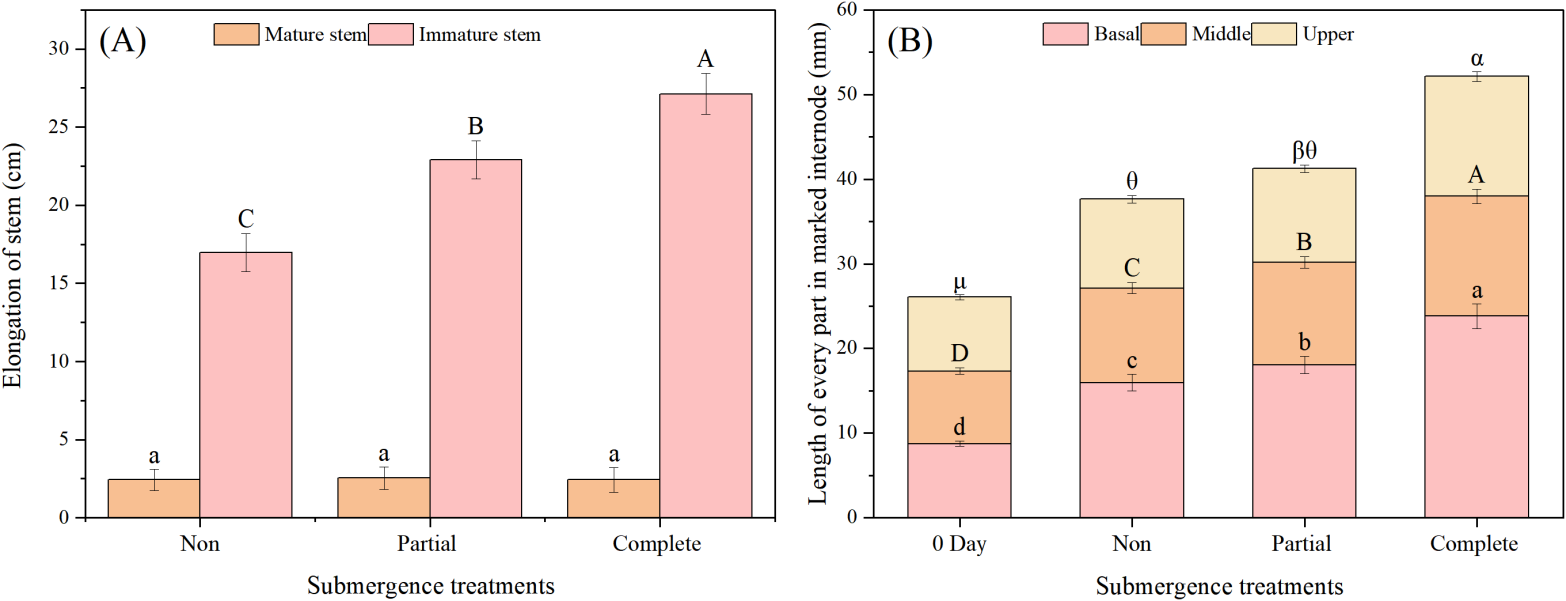
Elongation of mature and immature stems during treatment **(A)** and the length of every part in marked internode of *Alternanthera philoxeroides* before and after treatment **(B)**. Data are means ± SE, *n* = 20 (biological replicates, each representing an independent plant). 0 day indicates before treatment, other data represent measurements taken at 24 days after treatment. Different lowercase letters indicate significant differences among treatments (Duncan’s test, *p* < 0.05).

Stems were pre-classified into relatively mature and immature internodes. After treatment, relatively mature internodes exhibited a mean small growth increment (ca. 2.4 cm), with no significant differences across treatments (*p* > 0.05, **Figure 2A**). In contrast, immature internodes elongated significantly more than mature ones. These results indicate that submergence had negligible effects on mature internode elongation but strongly promoted immature internodes. Compared to the non-submergence group (16.98 cm), immature internode elongation increased significantly under both partial and complete submergence (*p* < 0.05, **Figure 2A**). Moreover, the mean immature internode elongation under complete submergence (27.12 cm) was significantly greater than that under partial submergence (22.89 cm) (*p* < 0.05, **Figure 2A**).

Marking and measuring the rapidly growing immature internodes of *A. philoxeroides* revealed that, relative to before-treatment (**Figure 2B****, Day 0**), the basal, middle, and upper regions of these internodes all elongated significantly after treatment (*p* < 0.05, **Figure 2B**), though their growth increments differed. Specifically, the basal region elongated more than the middle and upper regions (**Figure 2B**). In the basal region: both partial and complete submergence enhanced elongation compared to the non-submergence group (15.97 mm), with complete submergence (23.81 mm) inducing more growth than partial submergence (18.06 mm). In the middle region: elongation followed the order complete submergence > partial submergence > non-submergence (*p* < 0.05, **Figure 2B**). In the upper region: complete submergence induced significantly greater elongation than both the partial submergence group and the non-submergence group (*p* < 0.05, **Figure 2B**), while no significant difference was observed between partial submergence group and non-submergence group (*p* > 0.05, **Figure 2B**). These results indicate that complete submergence promoted elongation in all three regions (basal, middle, and upper), whereas partial submergence enhanced elongation mainly in the basal and middle regions, with negligible promoting effects on the upper region.

### 3.3 Newly formed internodes

Prior to the experiment, all *A. philoxeroides* plants had approximately 12.5 internodes. After 24 days of treatment, plants produced newly formed internodes under all treatments (**Table 2**). Specifically, the mean number of newly formed internodes was 2.95 in the partial submergence group, 2.30 in the complete submergence group, and 1.87 in the non-submergence group. Statistically, the number of newly formed internodes followed a clear order: partial submergence group > complete submergence group > non-submergence group, with significant differences among all treatments (*p* < 0.05, **Table 2**). These results indicate that partial submergence significantly promoted the formation of newly formed internodes in *A. philoxeroides*; while complete submergence resulted in fewer newly formed internodes than partial submergence.

**Table 2:** Internode number before and after treatments and number of newly formed internodes of *Alternanthera philoxeroides*.

Submergence treatments	Internode number before treatments	Internode number after treatments	Internode number of newly formed
Non-submergence	12.7 ± 0.28 a	14.6 ± 0.24 a	1.87 ± 0.09 c
Partial submergence	12.4 ± 0.23 a	15.4 ± 0.22 a	2.95 ± 0.08 a
Complete submergence	12.5 ± 0.26 a	14.8 ± 0.28 a	2.30 ± 0.13 b

Data are means ± SE, *n* = 20 (biological replicates, each representing an independent plant). Identical letters indicate no significant difference between treatments (Duncan’s test, *p* > 0.05); different letters indicate significant difference (Duncan’s test, *p* < 0.05).

### 3.4 Stem wall thickness and pith cavity area

**Figure 3:**
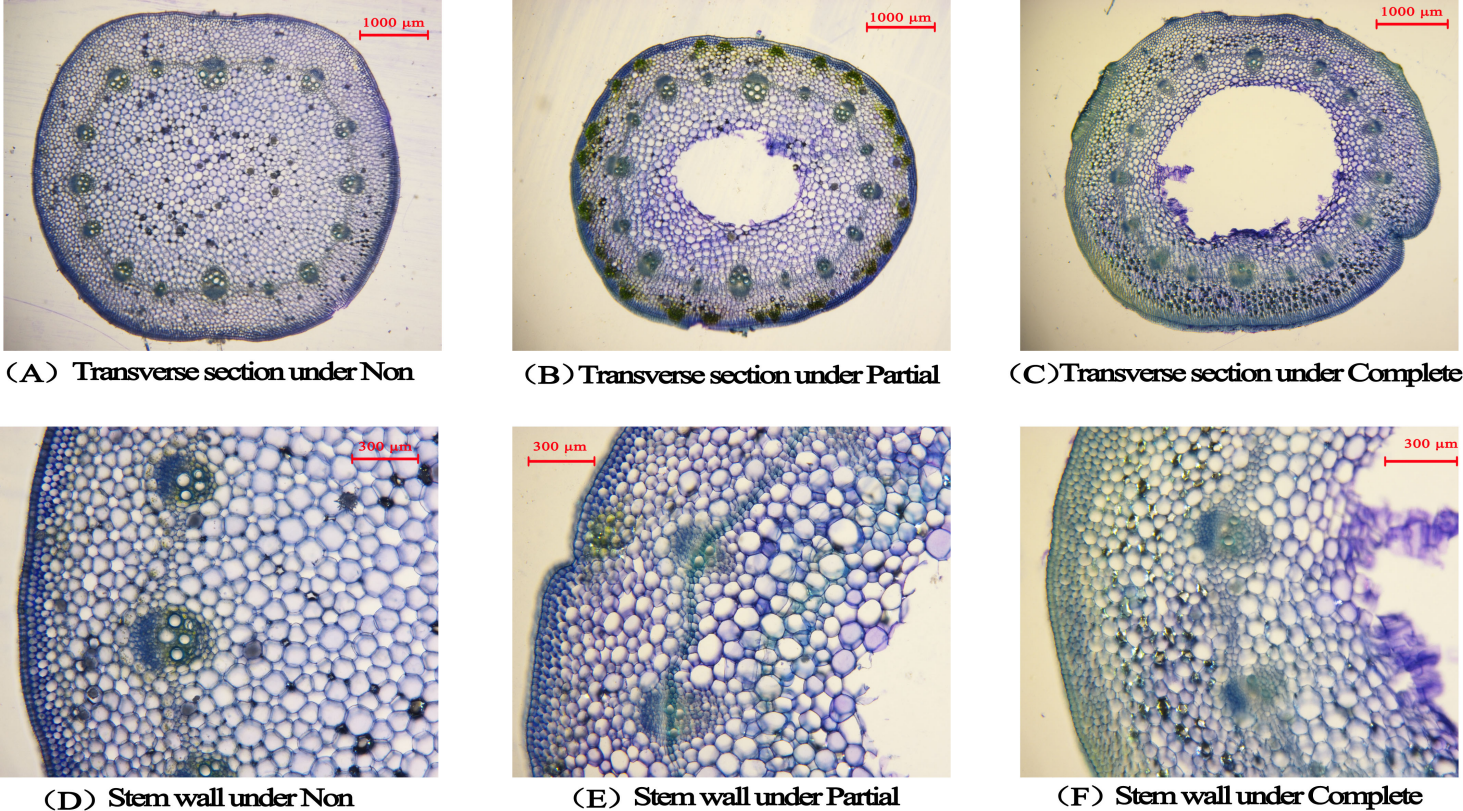
The transverse sections **(A)** was under non-submergence, **(B)** was under partial submergence and **(C)** was under complete submergence) and the stem wall thickness **(D)** was under non-submergence, **(E)** was under partial submergence and **(F)** was under complete submergence) in marked internode of *Alternanthera philoxeroides* after treatment (24 days). The middle blank area of the transverse sections represents the pith cavity area.

After 24 days of treatment, significant differences were observed in both stem wall thickness and pith cavity area at the base of immature internodes in *A. philoxeroides* (*p* < 0.05, **Table 3**, **Figure 3A–F**). Pith cavities formed under partial and complete submergence, but no pith cavities were detected in the non-submergence group. The pith cavity area under complete submergence was significantly larger than that under partial submergence (*p* < 0.05, **Table 3**). Concomitantly, both submergence treatments reduced stem wall thickness at the base of immature internodes; notably, the complete submergence group had significantly thinner walls than the partial submergence group and non-submergence group (*p* < 0.05, **Table 3**, **Figures 3A–F**). These results indicate that submergence (both partial and complete) induces pith cavity formation in *A. philoxeroides*, with complete submergence further promoting larger pith cavity areas and greater reductions in stem wall thickness compared to partial submergence.

**Table 3:** Stem wall thickness and pith cavity area of immature internodes of *Alternanthera philoxeroides* at the end of treatments.

Submergence treatments	Stem wall thickness (μm)	Pith cavity area (μm^2^)
Non-submergence	1456.35 ± 27.98 b	0
Partial submergence	1347.25 ± 64.26 b	756232.72 ± 8520.86 b
Complete submergence	1092.35 ± 86.75 a	1957822.37 ± 9650.69 a

Data are means ± SE, *n* = 3 (biological replicates, each representing an independent immature internode). Identical letters indicate no significant difference between treatments (Duncan’s test, *p* > 0.05); different letters indicate significant difference (Duncan’s test, *p* < 0.05).

### 3.5 Endogenous GA_4,_ ABA concentrations, and GA_4_/ABA ratio

**Figure 4:**
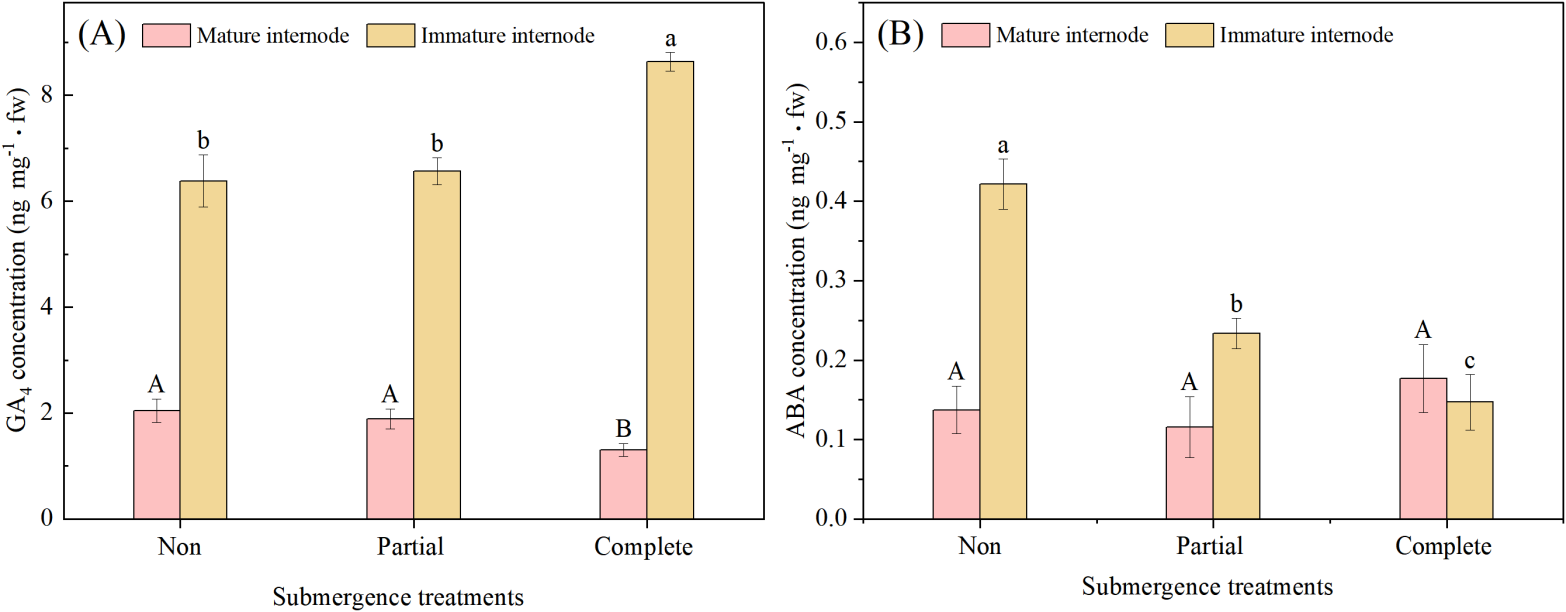
GA_4_**(A)** and ABA **(B)**concentration at the base of mature and immature internodes in *Alternanthera philoxeroides* after different treatments. Data are means ± SE, *n* = 3 biological replicates per treatment, with 4 plants pooled per biological replicate (tissue pooling was necessary for HPLC-MS/MS detection due to small tissue size). Identical letters indicate no significant difference between treatments (Duncan’s test, *p* > 0.05); different letters indicate significant difference (Duncan’s test, *p* < 0.05).

There was no significant difference in GA_4_ concentration at the base of mature internodes of *A. philoxeroides* between the non-submergence group and the partial submergence group, but both were significantly higher than that in the complete submergence group (*p* < 0.05, **Figure 4A**). By contrast, at the base of immature internodes, GA_4_ concentration was significantly higher under complete submergence than in the non-submergence group and partial submergence group (*p* < 0.05, **Figure 4A**), with no significant difference observed between the partial submergence group and the non-submergence group (*p* > 0.05, **Figure 4A**). These findings suggest that complete submergence promotes GA_4_ biosynthesis in immature internodes of *A. philoxeroides*.

ABA concentration at the base of mature internodes showed no significant differences among the three treatments. At the base of immature internodes, however, ABA concentration varied significantly with submergence intensity (*p* < 0.05, **Figure 4B**): it was highest in the non-submergence group, followed by the partial submergence, and lowest in the complete submergence group.

**Figure 5:**
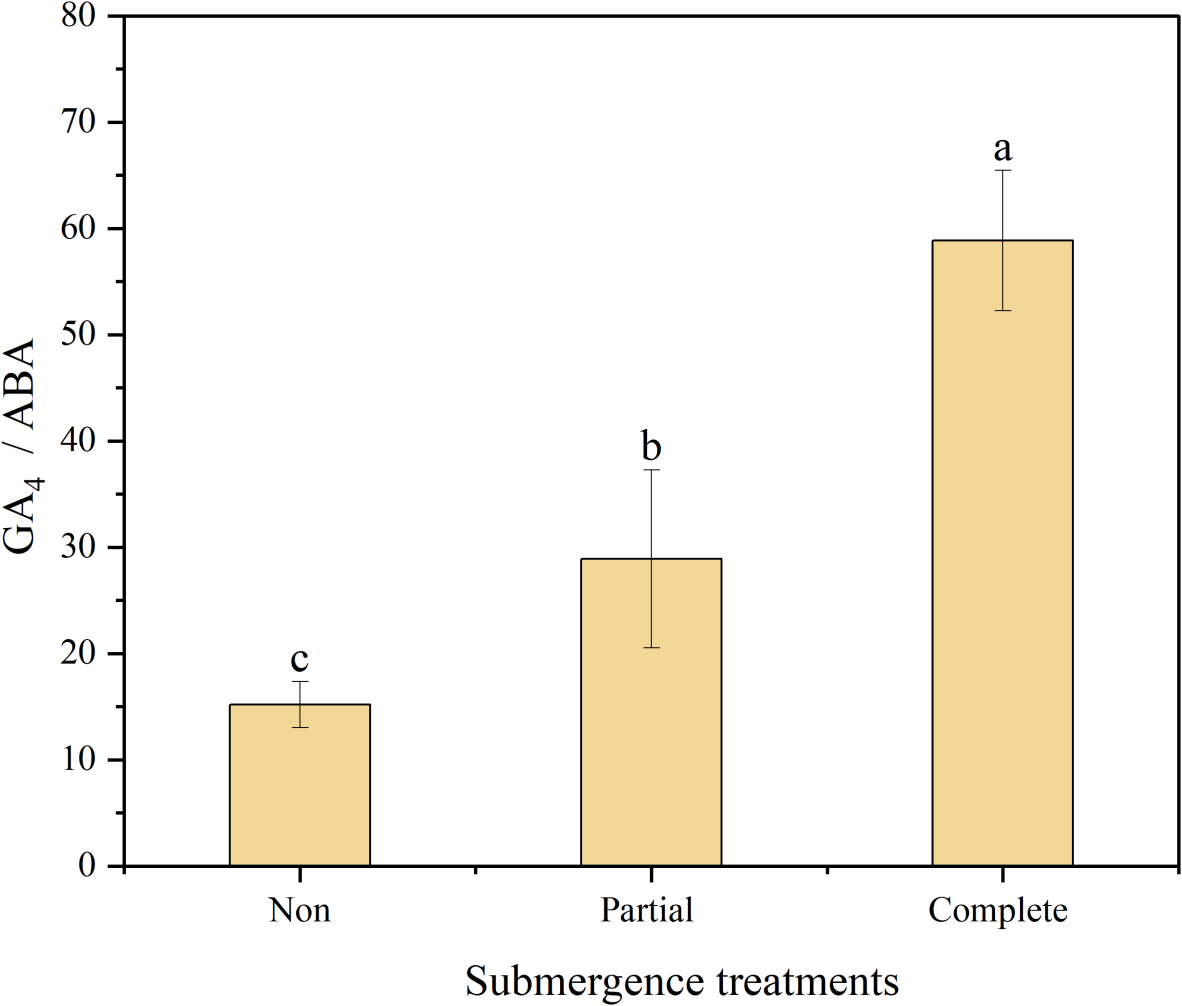
The concentration ratio of GA4/ABA at the base of immature internodes in *Alternanthera philoxeroides* after different treatments. Data are means ± SE, *n* = 3 biological replicates per treatment, with 4 plants pooled per biological replicate (tissue pooling was necessary for HPLC-MS/MS detection due to small tissue size). Different letters indicate significant difference (Duncan’s test, *p* < 0.05).

The GA_4_/ABA ratio differed significantly across the three treatments (*p* < 0.05, **Figure 5**). This ratio was highest under complete submergence, followed by partial submergence, and lowest under non-submergence, with significant distinct pairwise differences observed among all treatment groups (*p* < 0.05, **Figure 5**).

### 3.6 Relationships between pith cavity area, stem wall thickness, GA_4_/ABA concentrations, and the immature stem elongation

**Figure 6:**
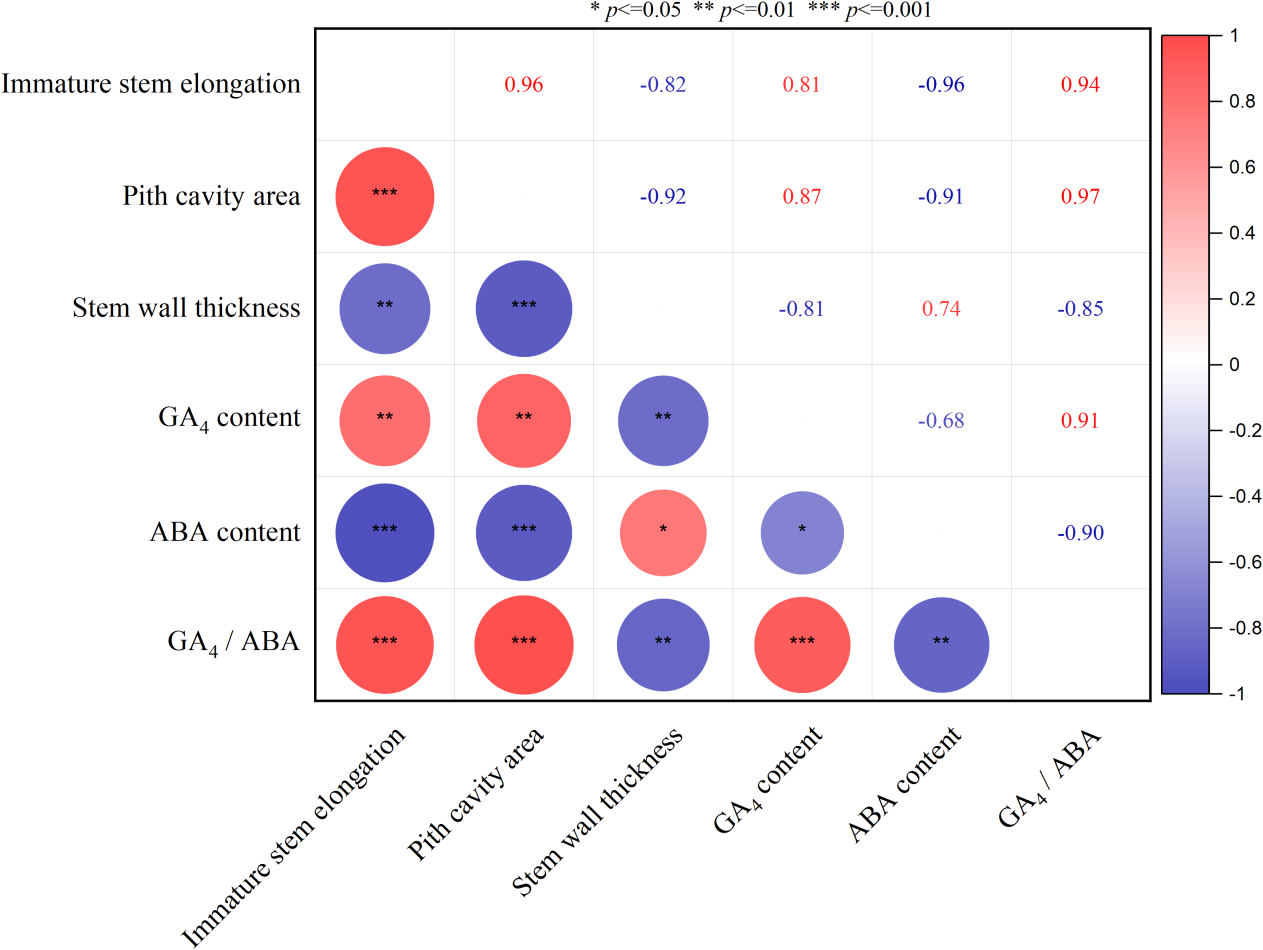
Correlations between pith cavity area, stem wall thickness, GA4/ABA concentrations, andimmature stem elongation. n = 9 independent biological samples (3 biological replicates per treatment × 3 hydrological regimes);for GA4/ABA quantification, each biological replicate consisted of pooled tissue from 4 plants (due to insufficient single-plant tissue for HPLC-MS/MSdetection).Red and blue circles indicate positive and negative correlations between variables, respectively; the larger the circle area, the stronger the correlation. *, **, and *** indicate significant correlations at *p* < 0.05, *p* < 0.01, and *p* < 0.001, respectively. All correlations were analyzed using Pearson correlation analysis.

Immature stem elongation of *A. philoxeroides* was significantly correlated with four key traits: pith cavity area, stem wall thickness, and endogenous GA_4_ and ABA concentrations (**Figure 6**). Specifically, an extremely significant positive correlation was observed between immature stem elongation and two traits: pith cavity area (r = 0.94, *p* < 0.001) and GA_4_ concentration (r = 0.91, *p* < 0.001). In contrast, it showed a significant negative correlation with stem wall thickness (r = -0.80, *p* < 0.01) and an extremely significant negative correlation with ABA concentration (r = -0.97, *p* < 0.001). Among the traits themselves: GA_4_ concentration displayed a significant negative correlation with ABA concentration (r = -0.85, *p* < 0.01), while also showing a highly significant positive correlation with pith cavity area (r = 0.87, *p* < 0.01). These interconnections imply two key regulatory links: (1) a potential synergism between pith cavity development and GA_4_ metabolism, and (2) an antagonistic relationship between GA_4_ and ABA—both of which may collectively modulate immature internode elongation under submergence stress (**Figure 6**).

## 4 Discussion

The responses of *A. philoxeroides* to submergence—investigated under three hydrological regimes (non-submergence, partial submergence, and complete submergence)—reveal a suite of adaptive traits that diverge markedly from conventional flood-tolerant strategies (e.g., the strategy of optimizing elongation under partial submergence in most wetland plants), offering critical insights into its invasive success across hydrologically dynamic landscapes. Notably, results revealed an unexpected growth pattern: complete submergence induces a significantly faster stem elongation than partial submergence, with this growth-promoting effect concentrated in immature internodes (especially their basal parts)—a pattern that contrasts with the typical strategy of prioritizing growth under milder submergence stress.

### 4.1 Ecological and physiological significance of key results: adaptive value of the unexpected stem elongation pattern

The most critical finding of this study is that complete submergence (rather than partial submergence) exerted a more significant promoting effect on stem elongation in *A. philoxeroides* (**Figures 1A, B**), and this growth-promoting effect was concentrated in immature internodes—particularly the basal parts (mean elongation: 23.81 mm, **Figures 2A, B**). This contrasts with the common understanding that “partial submergence optimizes elongation” in most wetland plants (Ashikari et al., 2025; Fukao et al., 2019). From an ecological adaptation perspective, this unexpected pattern can be explained as follows: under complete submergence, plants face extreme stress of “whole-plant hypoxia” and thus need to rapidly elongate stems to break through the water surface and restore gas exchange (Ashikari et al., 2025; Sasidharan et al., 2017). In contrast, partial submergence retains aerial tissues (upper stem segments) for oxygen uptake, alleviating hypoxia stress and thus reducing the pressure to elongate (Bailey-Serres et al., 2012; Yeung et al., 2018).

A further observation—no significant treatment differences in mature internode elongation, with immature internodes acting as the main contributors to total stem elongation—reflects a tissue-specific growth resources allocation strategy in *A. philoxeroides* (**Figures 2A, B**). Immature tissues (characterized by active cell division and elongation) are more sensitive to hormonal signals (e.g., GA_4_) and environmental stress, whereas mature tissues have highly lignified cell walls and low developmental plasticity, making them unresponsive to submergence-induced growth signals (Bashar et al., 2019; Yeung et al., 2018; Zhang et al., 2015). This position-specific response minimizes energy waste by avoiding unnecessary growth in non-adaptive tissues, ensuring that limited resources (e.g., carbohydrates, hormones) are concentrated on organs most critical for survival (immature internodes)—an important physiological basis for its adaptation to fluctuating hydrological environments (e.g., periodic flooding and drying).

### 4.2 Adaptive adjustments of anatomical traits: functional synergy between pith cavity enlargement and stem wall thinning

This study found that pith cavities formed at the base of immature internodes only under submergence treatments (partial/complete), with significantly larger pith cavity areas under complete submergence than under partial submergence (**Table 3**, **Figures 3A–F**). Concurrently, both submergence treatments reduced stem wall thickness at the base of immature internodes, and the complete submergence group exhibited significantly thinner walls than the partial submergence group and non-submergence group (**Table 3**, **Figures 3A–F**). The core function of this anatomical change is to enhance oxygen transport efficiency—a key adaptation to hypoxia under submergence (Bailey-Serres and Voesenek, 2008; Parlanti et al., 2011). As the core structure of aerenchyma, pith cavities form a continuous gas channel from the basal to upper part of the stem, reducing the diffusion resistance of oxygen within the stem and facilitating oxygen delivery to hypoxic tissues (Ayi et al., 2019; Fan et al., 2015). The larger pith cavity area under complete submergence can match the higher oxygen demand of rapid stem elongation, preventing necrosis of newly formed tissues due to hypoxia—a critical constraint for plants under severe submergence (Bailey-Serres et al., 2025; Voesenek and Bailey-Serres, 2013).

Stem wall thinning may be a concomitant result of pith cavity enlargement (e.g., plants prioritize parenchyma cell degradation for pith cavity formation over cell wall thickening) and, at the same time, reduces the mechanical resistance of the stem, providing structural flexibility for rapid internode elongation (Antonelli et al., 2019; Yang et al., 2019). Notably, no pith cavities were detected in the non-submergence group, indicating that pith cavity formation is a submergence-induced plastic response of *A. philoxeroides* rather than an inherent (constitutive) trait. This differs from the pattern of “basal pith cavity formation under non-submergence+ post-submergence enlargement” in wetland plants such as *Phragmites australis* (Cav.) Trin (Daniel and Hartman, 2024), reflecting divergent strategies of anatomical plasticity among different wetland species.

### 4.3 Interconnections between anatomical traits, hormonal regulation, and immature internode elongation

The significant correlations observed between immature internode elongation and key traits—pith cavity area, stem wall thickness, and GA_4_/ABA concentrations—provide critical insights into the integrated adaptive strategy of *A. philoxeroides* under submergence (**Figure 6**). These interconnections are not merely coincidental but reflect a coordinated regulatory network, yet extends, existing understanding of flood adaptation in wetland plants (Cox et al., 2004; Zou et al., 2019). Specially, complete submergence induces significant GA_4_ accumulation in the basal parts of *A. philoxeroides’* immature internodes (**Figure 4A**)—the region with highly active cell division and elongation—resulting in a strongly significant positive correlation between GA_4_ concentration and immature internode elongation (r = 0.91, *p* < 0.001; **Figure 6**). This aligns with well-established roles of gibberellins in promoting stem elongation under submergence (Ashikari et al., 2025; Ayano et al., 2014; Zou et al., 2019). In immature tissues, submergence stress may activate the expression of GA synthesis genes (e.g., GA3oxidase) while inhibiting degradation genes (e.g., GA20oxidase), leading to the accumulation of bioactive GA_4_ (Ashikari et al., 2025; Ayano et al., 2014). Notably, the tissue-specific distribution pattern of GA_4_ in *A. philoxeroides* under submergence aligns closely with that of deepwater rice, a classic flood-tolerant crop (Ashikari et al., 2025; Ayano et al., 2014). Deepwater rice also exhibits targeted GA accumulation in the intercalary meristems of young internodes under submergence, where GA activates cell elongation-related genes to drive rapid internode elongation, enabling the plant to escape hypoxia by reaching the water surface (Ashikari et al., 2025; Fukao et al., 2019; Yin et al., 2017). The decrease in GA_4_ in mature tissues may reflect resource redistribution in plants: by inhibiting GA signaling in mature tissues, plants reduce unnecessary growth and prioritize resources to support the elongation of immature tissues.

Importantly, a significant positive correlation was also observed between GA_4_ and pith cavity area (r = 0.87, *p* < 0.01) (**Figure 6**). This suggests a “GA_4_-mediated synergistic regulatory mechanism”: under complete submergence, GA_4_ not only directly promotes the elongation of immature cells but also may induce pith cavity enlargement by regulating genes related to pith cavity formation (e.g., genes regulating programmed cell death). Together, these two processes form an adaptive loop of “rapid elongation + oxygen guarantee”, resolving the contradiction of “elongation requiring energy/oxygen while submergence causing oxygen deficiency” (Ashikari et al., 2025). The antagonistic relationship between GA_4_ and ABA (r = -0.85, *p* < 0.01) further reinforces this network. ABA is known to repress growth under stress to conserve resources (Azuma et al., 1995; Lorrai et al., 2018). Our data show that complete submergence reduces ABA levels and promotes GA_4_ accumulates, creating a hormonal environment favoring elongation. This GA_4_-ABA balance mirrors patterns in Oryza sativa under submergence, where ABA degradation is prerequisite for GA-mediated elongation (Vriezen, 2003), suggesting a conserved hormonal crosstalk module that *A. philoxeroides* has co-opted for its invasive strategy.

## 5 Conclusion

This study investigated the adaptive responses of the wetland plant *A. philoxeroides* to three hydrological regimes (non-submergence, partial submergence, and complete submergence), focusing on the anatomical, hormonal, and growth mechanisms underlying its flood tolerance. Complete submergence triggered greater stem elongation than partial submergence, with this effect concentrated in the bases of immature internodes. Submergence further induced specific anatomical remodeling at these bases: pith cavity formation and reduced stem wall thickness, while concurrently promoting GA_4_ accumulation and reducing ABA levels here. Collectively, the coordinated interplay of targeted growth plasticity, submergence-specific anatomical changes, and tissue-specific GA_4_-ABA balance constitutes the core adaptive mechanism enabling *A. philoxeroides* to invade and thrive in flood-prone ecosystems. This mechanism explains the species’ ecological success as an invasive wetland species and provides a novel model for understanding “hormone-anatomy-growth” synergy in flood adaptation.

## Data Availability

The original contributions presented in the study are included in the article/**Supplementary Material**. Further inquiries can be directed to the corresponding author.
